# Dorsal Raphe 5-HT Neurons Utilize, But Do Not Generate, Negative Aversive Prediction Errors

**DOI:** 10.1523/ENEURO.0132-21.2022

**Published:** 2022-02-17

**Authors:** Rachel A. Walker, Rebecca L. Suthard, Taylor N. Perison, Nora M. Sheehan, Caitlin C. Dwyer, Jillian K. Lee, Eghosa K. Enabulele, Madelyn H. Ray, Michael A. McDannald

**Affiliations:** Department of Psychology and Neuroscience, Boston College, Chestnut Hill, MA 02467

**Keywords:** discrimination, extinction, fear, prediction error, serotonin, Tph2-cre

## Abstract

The dorsal raphe nucleus (DRN) contains the largest population of serotonin (5-HT) neurons in the central nervous system. 5-HT, synthesized via tryptophan hydroxylase 2 (Tph2), is a widely functioning neuromodulator implicated in fear learning. Here, we sought to investigate whether DRN 5-HT is necessary to reduce fear via negative prediction error (–PE). Using male and female TPH2-cre rats, DRN^tph2+^ cells were selectively deleted via cre-caspase (rAAV5-Flex-taCasp3-TEVp) in experiment 1. Rats then underwent fear discrimination during which three cues were associated with unique foot shock probabilities: safety *p* = 0.00, uncertainty *p* = 0.375, and danger *p* = 1.00. Rats then received selective extinction to the uncertainty cue, a behavioral manipulation designed to probe –PE. Deleting DRN^tph2+^ cells had no impact on initial discrimination but slowed selective extinction. In experiment 2, we used a within-subjects optogenetic inhibition design to causally implicate DRN^tph2+^ cells in prediction error signaling. Male and female TPH2-cre rats received intra-DRN infusions of cre-dependent halorhodopsin (rAAV5-Ef1a-DIO-eNpHR3.0-eYFP) or cre-YFP. DRN^tph2+^ cells were inhibited specifically during the time of prediction error or a control period. Illumination during either positive prediction error (+PE) or control periods had no impact on fear to the uncertainty cue. Inhibition of DRN^tph2+^ cells at the time of –PE did not impact immediate fear, but facilitated selective extinction in postillumination sessions. Together, these results demonstrate a role for DRN^tph2+^ cells in using, but not generating, –PE to weaken cue-shock associations.

## Significance Statement

Uncertainty is pervasive in life, and responding appropriately and proportionally to uncertain threats is critical for adaptive behavior. Aversive prediction errors provide an updating mechanism to generate appropriate fear responses amid uncertainty. Particularly, negative prediction errors (–PEs) are crucial signals for decreasing fear, but where and how the brain generates these signals is unknown. Our results demonstrate that dorsal raphe tryptophan hydroxylase 2 (tph2)+ cells, a marker for serotonergic neurons, are not the source of –PE, but these neurons receive and use the error to carry out fear updating. Understanding the neural network responsible for aversive prediction errors will inform the neurologic basis of fear and may also provide insights into disorders, such as posttraumatic stress disorder and anxiety disorders, characterized by excessive/inappropriate fear.

## Introduction

Aversive prediction errors are critical learning signals that allow for calibration of fear responses in the face of uncertainty. Prediction errors may be directionally positive or negative depending on whether threat was initially underestimated or overestimated, respectively. Of particular relevance to the present study, a negative prediction error (–PE) occurs when a predicted outcome is worse than the actual outcome received (e.g., expecting a foot shock, but receiving none) and acts to weaken the cue-outcome association (Rescorla, 1970). Because –PEs decrease fear to predictive cues on future encounters, they are essential for the display of appropriate, adaptive fear responses and warrant investigation into how they are generated in the brain.

While recent evidence has identified the ventrolateral periaqueductal gray (vlPAG) as the source of aversive positive prediction error (+PE) generation (McNally and Cole, 2006; Johansen et al., 2010; Walker et al., 2020), the neural mechanisms underlying aversive –PEs are currently unknown. A previously suggested site of –PE generation is the dorsal raphe nucleus (DRN). One review hypothesized that DRN serotonin (5-HT) may be the locus of an aversive PE based on known function and anatomic substrates (Daw et al., 2002), and others have suggested the DRN may even be involved in positive or unsigned prediction errors (Matias et al., 2017). DRN 5-HT neurons also show activation because of punishment (Cohen et al., 2015). However, only one previous study has experimentally tested DRN involvement in prediction error. During fear conditioning, neurotoxic lesions of the DRN were shown to prevent decreases in fear to an uncertain cue (Berg et al., 2014). Further, DRN lesions resulted in reduced fear extinction of a deterministic cue compared with controls. Because of the impaired ability to decrease fear, these findings suggested disrupted –PE signaling as the source of these effects. The evidence suggesting DRN-generation of –PEs was not tied to the error period nor 5-HT neurons, however, because of the nature of the lesion. Given the DRN’s largest cell population is serotonergic (Steinbusch, 1981) and 5-HT has been shown to play a role in fear learning and expression (Grahn et al., 1999; Schweimer and Ungless, 2010; McDevitt et al., 2011; Spannuth et al., 2011; Li et al., 2016; Ren et al., 2018; Sengupta and Holmes, 2019), it was hypothesized that this subset of neurons in the DRN generate aversive –PEs.

Here, we sought to uncover a relationship between DRN serotonergic –PE activity and fear updating. To do so, we employed a fear discrimination procedure in which a safety cue predicted shock omission deterministically and an uncertainty cue predicted shock omission probabilistically (Berg et al., 2014; Wright et al., 2015; Walker et al., 2018, 2020). Fear to the uncertainty cue was of particular interest, as this behavior would be reliant on –PE updating. In experiment 1, DRN cells expressing the serotonergic marker tryptophan hydroxylase 2 (tph2; DRN^tph2+^) were selectively deleted to determine whether this impacts fear discrimination or extinction of the uncertain cue. In experiment 2, DRN^tph2+^ activity was selectively inhibited around the time of predicted (safety) and surprising (uncertainty) foot shock omission. Inhibition during shock periods in separate sessions was used to test for possible involvement in positive or unsigned PE signaling. Analyses focused on subsequent changes in fear to the safety and uncertainty cues and the temporal emergence of these changes. Our findings indicate that DRN^tph2+^ neurons do not generate –PEs, rather, this population likely receives the –PE signal to carry out fear updating effects.

## Materials and Methods

### Experimental subjects

All rats were maintained on a 12/12 h light/dark cycle (lights on 6 A.M. to 6 P.M.). Rats were single housed, first given *ad libitum* access to standard laboratory chow (18% Protein Rodent Diet #2018, Harlan Teklad Global Diets) then food restricted to 85% of their free-feeding body weight before Pavlovian fear conditioning. Water was available *ad libitum* in the home cage. Dustless Precision Test Pellets (Bio-Serv, catalog #F0021) were used in the experimental chambers. All protocols were approved by the Boston College Animal Care and Use Committee, and all experiments were conducted in accordance with the National Institutes of Health guidelines regarding the care and use of rats for experimental procedures.

For experiment 1, subjects were 14 female and 9 male adult TPH2-cre transgenic rats on the background of Long–Evans born in the laboratory. All rats underwent stereotaxic surgery under isoflurane (Henry Schein Medical) anesthesia. Rats received 0.75-μl bilateral infusions of a 50/50 mixture of cre-caspase (rAAV5-Flex-taCasp3-TEVp) and cre-YFP (rAAV5-Ef1a-DIO-eYFP) to delete 5-HT neurons (Caspase; *n* = 12; 7 females) or cre-YFP only (YFP; *n* = 11; 7 females) in the DRN (−8.00 AP, ±0.40 ML, −6.45 DV from skull). 10 min elapsed before the syringe was withdrawn to allow for viral diffusion. Rats received at least 10 d of undisturbed recovery postsurgery before beginning behavior.

For experiment 2, subjects were 14 female and 6 male adult TPH2-cre transgenic rats on the background of Long–Evans born in the laboratory. All rats underwent stereotaxic surgery under isoflurane anesthesia. Rats received 0.75-μl bilateral infusions of cre-dependent halorhodopsin (rAAV5-Ef1a-DIO-eNpHR3.0-eYFP; *n* = 10; 7 females) or cre-YFP (rAAV5-Ef1a-DIO-eYFP; *n* = 10; 7 females) in the DRN (−8.00 AP, ±0.40 ML, −6.45 DV from skull). Ten minutes elapsed before the syringe was withdrawn to allow for viral diffusion. Fiber optic ferrules were bilaterally implanted in the DRN (−8.10 AP, ±1.83 ML, −6.35 DV from skull at ±10° angle) to permit 532-nm light illumination. Implants were secured with dental cement surrounded by a cut 50 ml plastic centrifuge tube to protect the implants. Rats received two weeks of undisturbed recovery postsurgery with prophylactic antibiotic treatment (cephalexin; Henry Schein Medical) before resuming behavior. In order to be considered for analysis, rats had to maintain a nose poke rate higher than five pokes per minute. One rat was excluded from analyses based on nose poke criteria.

### Apparatus

The apparatus for Pavlovian fear discrimination consisted of eight, individual sound-attenuated enclosures that each housed a behavior chamber with aluminum front and back walls, clear acrylic sides and top, and a metal grid floor. Each grid floor bar was electrically connected to an aversive shock generator (Med Associates). A single food cup and a central nose poke opening, equipped with infrared photocells, were present on one wall. Auditory stimuli were presented through two speakers mounted on the ceiling of each sound-attenuated enclosure. Behavior chambers were modified to allow for free movement of the optical cables during behavior; plastic funnels were epoxied to the top of the behavior chambers with the larger end facing down, and the tops of the chambers were cut to the opening of the funnel. Green (532 nm, 500 mW) lasers (Shanghai Laser & Optics Century Co, Ltd.) were used to illuminate the DRN. Optical cables were connected to the lasers via 1 × 2 fiber optic rotatory joints (Doric). Rats were bilaterally connected to the optical cables by a ceramic sleeve placed over the implanted ferrule and ceramic ferrule end of the cable. Black shrink-wrap was also placed on the ends of the cables to block light emission into the behavioral chamber. A PM160 light meter (Thorlabs) was used to measure light output.

### Nose poke acquisition

Before behavioral testing began, all rats were given 2 d of preexposure in the home cage to the pellets used for rewarded nose poking. Rats were then shaped to nose poke for these pellets in the experimental chamber. During the first session, rats were issued one pellet every 60 s with the nose poke port removed for 30 min. Rats were then issued pellets on a fixed ratio schedule in which one nose poke yielded one pellet until they reached at least 50 nose pokes (FR1) in a session. Over the next 5 d, rats were reinforced for nose pokes on a variable interval schedule first on average every 30 s (VI-30), for one session, then on average every 60 s (VI-60), for four sessions. All subsequent conditioning sessions were run with a background VI-60 reinforcement schedule that was completely independent of auditory cue or foot shock presentation on conditioning trials. Rats in experiment 2 were trained through four VI-60 sessions and then underwent surgery and recovery before receiving two reminder VI-60 sessions and beginning preexposure.

### Preexposure

In two separate sessions, each rat was preexposed to the three 10-s auditory cues to be used in Pavlovian fear discrimination. These 42 min sessions consisted of four presentations of each cue (12 total presentations) with a mean intertrial interval (ITI) of 3.5 min. The order of trial type presentation was randomly determined by the behavioral program and differed for each rat during each session. Auditory cues consisted of repeating motifs of: broadband click, phaser, or trumpet.

### Fear discrimination

Every session began with a 5-min habituation period, and ITIs were 3.5 min on average. Each cue was associated with a unique probability of foot shock (0.5 mA, 0.5 s): danger, *p* = 1.00; uncertainty, *p* = 0.25; and safety, *p* = 0.00. Cue identity was counterbalanced within groups. Foot shock was administered 2 s following the termination of the auditory cue on danger and uncertainty-shock trials. There were 16 total trials per session: four danger trials, 6 uncertainty-omission trials, two uncertainty-shock trials, and four safety trials. The trial type order was randomly determined by the behavioral program, and differed for each rat, each session for both experiments. Rats in experiment 1 underwent 16 sessions of Pavlovian fear discrimination before moving on to selective extinction. Rats in experiment 2 underwent 12 sessions of Pavlovian fear discrimination before undergoing optogenetic manipulations. During the last two fear discrimination sessions (11–12), rats were connected to “dummy” cables like those used during the optogenetic manipulation, but that did not deliver light, to habituate them to the cables.

### Selective extinction

During selective extinction sessions, the uncertainty cue was no longer associated with foot shock. However, the foot shock probability associated with danger and safety cues remained the same as in fear discrimination: danger, *p* = 1.00; uncertainty, *p* = 0.00; and safety, *p* = 0.00. The order of trial type presentation was randomly determined by the behavioral program, and differed for each rat, each session for both experiments. Rats in experiment 1 received eight sessions of selective extinction following their 16 fear discrimination sessions. There were 18 total trials per session consisting of four danger trials, eight uncertainty trials, and four safety trials. Rats in experiment 2 received 8 total sessions of selective extinction, the first four during the –PE optogenetic manipulation and the second four without any additional manipulation or cables present. There were 18 total trials per session consisting of four danger trials, eight uncertainty trials, and six safety trials.

### Optogenetic manipulations

Rats in experiment 2 underwent eight total sessions, split in two sets of 4, of optogenetic manipulation. The first four sessions consisted of a +PE optogenetic manipulation occurring immediately following fear discrimination training. After the +PE optogenetic manipulation, the rats received an additional two fear discrimination sessions before beginning the –PE optogenetic manipulation (second set of four sessions) to wash out any potential carry over effects of the initial light manipulation.

For both manipulations, optical inhibition was achieved via delivery of 25 mW of 532-nm “green” light on each side. Light was produced by a DPSS laser connected to an optical commutator attached to a custom-made behavioral cable (Multimode Fiber, 0.22 NA, High-OH, Ø200 μm Core), which connected to the implanted optical ferrule (2.5 mm OD, 230 μm Bore Multimode Ceramic Zirconia). Light output of 25 mW was chosen based on calculations the optical fibers will produce ∼5 mW/mm^2 of light at distance of 1.2 mm from fiber tip.

For the +PE optogenetic manipulation, the DRN was illuminated during the foot shock on uncertainty-shock and danger trials. Illumination on both uncertainty and danger trials occurred for 4 s, beginning immediately after auditory cue offset (2 s), continuing during foot shock (0.5 s), and ending 1.5 s after foot shock offset. During the –PE optogenetic manipulation, the uncertainty cue was now selectively extinguished as described above. The DRN was illuminated during the omission period on uncertainty and safety trials. Illumination on both uncertainty and safety trials occurred for 4 s, beginning immediately after auditory cue offset.

### Histology

Rats were deeply anesthetized using isoflurane and perfused with 0.9% biological saline and 4% paraformaldehyde in a 0.2 m potassium phosphate buffered solution. Brains were extracted and postfixed in a 10% neutral-buffered formalin solution for 24 h, stored in 10% sucrose/formalin, frozen at −80°C and sectioned via a sliding microtome. Brains were processed for fluorescent microscopy. Tissue was processed with fluorescent anti-tryptophan hydroxylase immunohistochemistry and NeuroTrace (ThermoFisher Scientific) to ensure deletion of DRN^tph2+^ neurons (experiment 1) or transfection of DRN^tph2+^ neurons (experiment 2). This tissue was mounted on glass slides with VECTASHIELD HardSet Antifade Mounting Medium (Vector Laboratories). Deletion extent, viral transfection, and optical implant sites were confirmed by comparison to a rat brain atlas (Paxinos and Watson, 2007).

### Baseline nose poke analyses

The time stamp for every nose poke and event onset (cues and shocks) during each session was recorded automatically. Raw data were processed in MATLAB to extract nose poke rates during three periods: the baseline, which was 20 s before cue onset; the 10-s cue; and the postcue period, which was 4 s following cue offset. Baseline nose pokes are reported in pokes/min and analyzed with ANOVA.

### Calculating and analyzing suppression ratios

Suppression of rewarded nose poking was used as the behavioral indicator of fear. Nose poke rates were calculated for two temporal windows. A suppression ratio for total cued fear was calculated from nose poke rates during a 20-s baseline period just before cue onset and the 10-s cue period: (baseline – cue/baseline + cue). Complete nose poke suppression was signified by a suppression ratio of “1.00” during the cue relative to baseline, indicating a high level of fear. No nose poke suppression was signified by a suppression ratio of “0.00,” indicating no fear. Intermediate values indicated graded levels of fear.

### Session-by-session analyses

In experiment 1, repeated-measures ANOVA for suppression ratios with between factors of group (YFP vs Caspase) and sex (female vs male), plus within factors of session (2 preexposure and 16 discrimination) and cue (danger vs uncertainty vs safety) were used compare behavior during fear discrimination. Similar ANOVAs over the last day of discrimination plus eight sessions of selective extinction (nine total sessions) compared fear levels to each cue to determine the effects of DRN^tph2+^ deletion during selective extinction.

In experiment 2, repeated-measures ANOVA for suppression ratios with between factors of group (YFP vs eNpHR) and sex (female vs male), plus within factors of session (2 preexposure and 12 discrimination) and cue (danger vs uncertainty vs safety) were used compare behavior during fear discrimination. Similar ANOVAs were run for the +PE optogenetic manipulation and –PE optogenetic manipulation/selective extinction sessions to determine the impact of light illumination.

### Trial-by-trial analyses

In order to determine whether changes in fear were because of within-session or between-session fear updating, the first selective extinction session postoptogenetics was isolated. The uncertainty cue was no longer paired with shock on any trials in this session. Six uncertainty cue trials were sampled during this session to look at fear at the trial level. Repeated-measures ANOVA for suppression ratios with between subjects factor of group (YFP vs eNpHR) and within factor of trial was used to compare fear within-session.

## Results

### Experiment 1

#### Baseline nose poke rates

**Figure 1.**
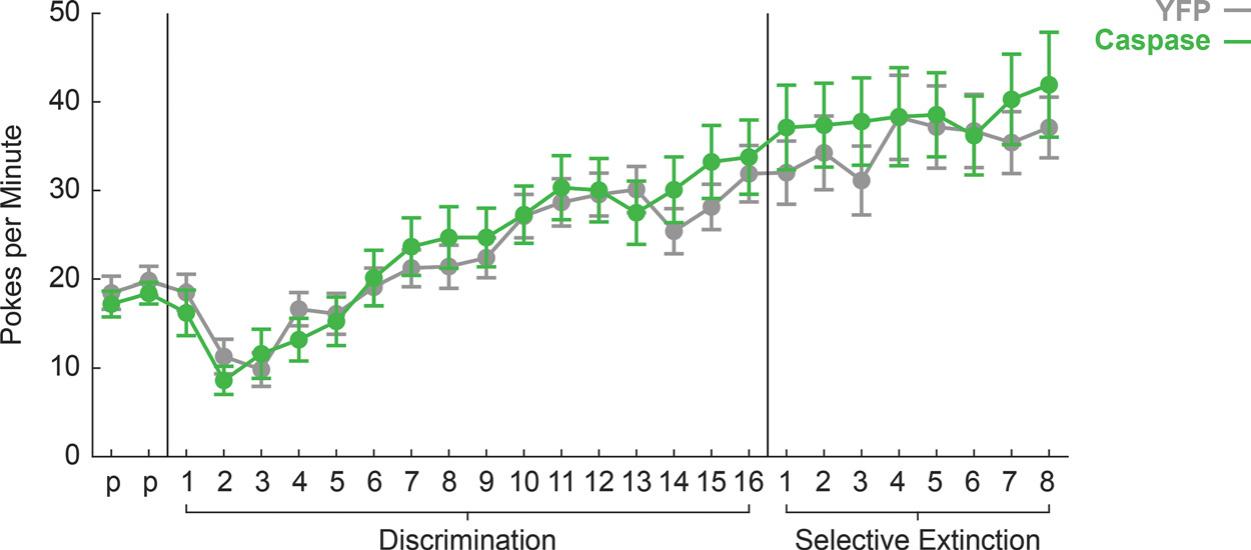
YFP versus Caspase baseline nose poke rates. Mean ± SEM baseline nose poke rates for YFP (gray) and caspase rats (green) are plotted for the two preexposure (p), 16 discrimination, and eight selective extinction sessions. No group differences were detected in baseline nose poke behavior.

Information for primary statistical results is provided in Table 1. All rats readily nose poked for reward, and no effect of DRN^tph2+^ deletion was observed. Repeated-measures ANOVA for baseline nose poke rate (within factor: session; between factors: sex and group) revealed main effects of session (*F*_(25,475)_ = 31.57, *p* < 0.001, η^2^p = 0.62, power = 1.00^a^), sex (*F*_(1,19)_ = 23.20, *p* < 0.001, η^2^p = 0.55, power = 0.99^a^), and a sex × session interaction (*F*_(25,475)_ = 6.84, *p* < 0.001, η^2^p = 0.27, power = 1.00^a^). Sex effects were driven by higher poke rates in males compared with females, an effect consistent with previous behavioral findings in this paradigm (Walker et al., 2018, 2020). Importantly, there were no effects or interactions with group throughout behavioral testing (all *F* < 0.91, all *p* > 0.05), indicating YFP and Caspase groups poked at similar rates (Fig. 1).

**Table 1:** Statistical table

	Data structure	Type of test	Power
a	Normal distribution	Repeated-measures ANOVA	1.00
b	Normal distribution	Repeated-measures ANOVA	1.00
c	Normal distribution	Repeated-measures ANOVA	0.82
d	Normal distribution	Repeated-measures ANOVA	1.00
e	Normal distribution	Repeated-measures ANOVA	1.00
f	Normal distribution	Repeated-measures ANOVA	1.00
g	Normal distribution	Repeated-measures ANOVA	1.00
h	Normal distribution	Repeated-measures ANOVA	0.52
i	Normal distribution	Independent samples *t* test	[−0.35, −0.026]
j	Normal distribution	Independent samples *t* test	[−0.19, 0.14]
k	Normal distribution	Repeated-measures ANOVA	0.20

#### Deletion of DRN^tph2+^ neurons does not impact fear discrimination

If DRN 5-HT neurons are required for –PE signaling, then deletion of these neurons would be expected to interfere with fear discrimination, particularly in the ability to decrease fear to the probabilistic, uncertainty cue. By contrast, deletion would not be expected to interfere with expression of high levels of fear to a deterministic cue, like that usually seen to a danger cue.

**Figure 2.**
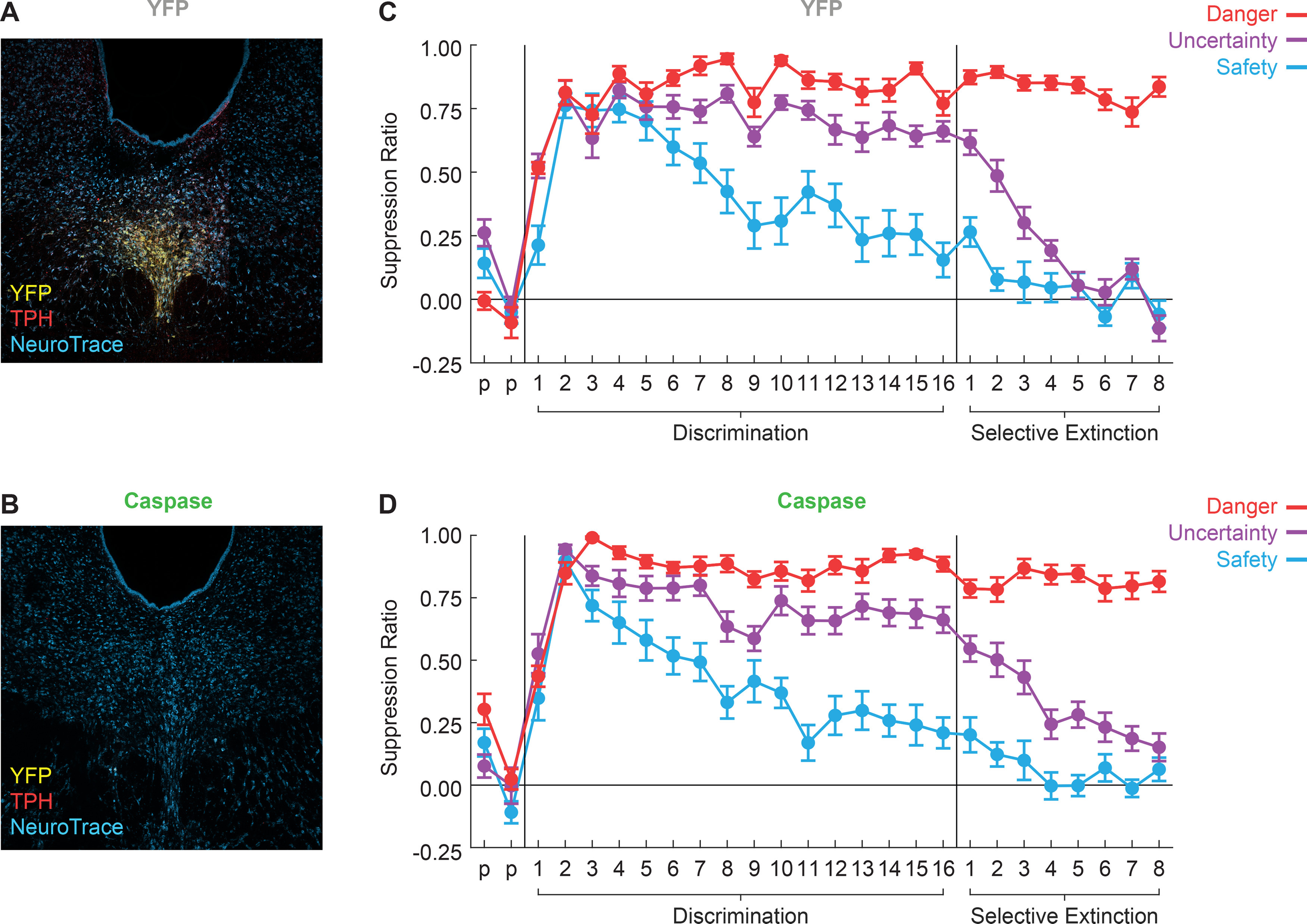
DRN^tph2+^ deletion does not impact discrimination but impairs extinction of uncertainty. ***A***, Representative image shows cre-YFP (yellow) labeling of neurons in the DRN against tph2 immunohistochemistry (red) in a TPH2-cre rat. Fluorescent Nissl staining (NeuroTrace) shows neuronal cell bodies (blue). ***B***, Representative image shows DRN^tph2+^ deletion via cre-caspase with labeling as in ***A***. ***C***, Mean ± SEM suppression ratios for YFP rats to danger (red), uncertainty (purple), and safety (blue) are shown for the two preexposure sessions (p), 16 discrimination sessions, and eight selective extinction sessions. ***D***, Caspase suppression ratio data shown as in ***A***.

While rats in the YFP group showed tph2+ cells intact (Fig. 2*A*), all rats in the Caspase group showed robust deletion of tph2+ cells throughout the DRN, such that no tph2+ cells could be detected in the DRN when stained with anti-tph2 immunohistochemistry (Fig. 2*B*). Rats in both groups showed low/no suppression to any cue during the two preexposure sessions. As expected, rats in the YFP group with DRN^tph2+^ cells intact showed the typical pattern of behavior during discrimination sessions. Fear was initially generalized across all cues before becoming selective as the rats learned to discriminate, showing high fear to danger, intermediate fear to uncertainty, and low fear to safety (Fig. 2*C*). Somewhat surprisingly, the Caspase group demonstrated a similar pattern of fear behavior during discrimination (Fig. 2*D*). These results were confirmed by ANOVA finding no effects of or interactions with group during preexposure or discrimination (all *F* < 1.90, all *p* > 0.05), but significant effects of cue (*F*_(2,38)_ = 48.86, *p* < 0.001, η^2^p = 0.72, power = 1.00^b^), session (*F*_(15,285)_ = 14.92, *p* < 0.001, η^2^p = 0.44, power = 1.00 ^b^), and a cue × session interaction (*F*_(30,570)_ = 6.91, *p* < 0.001, η^2^p = 0.27, power = 1.00 ^b^). A main effect of sex was also present during discrimination sessions (*F*_(1,19)_ = 4.49, *p* = 0.048, η^2^p = 0.19, power = 0.52 ^b^) because of higher average suppression ratios in females (mean = 0.72) compared with males (mean = 0.58). These findings demonstrated that both YFP and Caspase rats learned to differentiate the three auditory cues and the two groups began selective extinction with equivalent levels of fear discrimination.

#### Deletion of DRN^tph2+^ neurons impairs extinction of uncertainty

After fear discrimination, all rats underwent eight sessions during which the uncertainty cue was now selectively extinguished. YFP rats extinguished fear to the uncertainty cue across these sessions, resulting in a fear level equivalent to that of the safety cue (Fig. 2*C*). Caspase rats, however, extinguished fear to uncertainty more slowly and did not achieve the same level of extinction as the YFP group (Fig. 2*D*). These results were supported by a repeated-measures ANOVA demonstrating a significant session × group interaction (*F*_(8,152)_ = 2.07, *p* = 0.042, η^2^p = 0.098, power = 0.82^c^) for the uncertainty cue. A session × sex interaction was also present (*F*_(8,152)_ = 2.58, *p* = 0.011, η^2^p = 0.12, power = 0.91^c^) during these sessions. There were no group differences in fear to the danger or safety cues during selective extinction (all *F* < 2.82, all *p* > 0.05), as expected since they maintained their contingencies. While DRN^tph2+^ deletion did not impact fear during discrimination, these results demonstrate DRN^tph2+^ neurons are necessary for accurate extinction, which could mean they are involved in a –PE signal. In experiment 2, we wanted to explicitly test whether DRN^tph2+^ neurons generate or use –PEs.

### Experiment 2

#### Baseline nose poke rates

**Figure 3.**
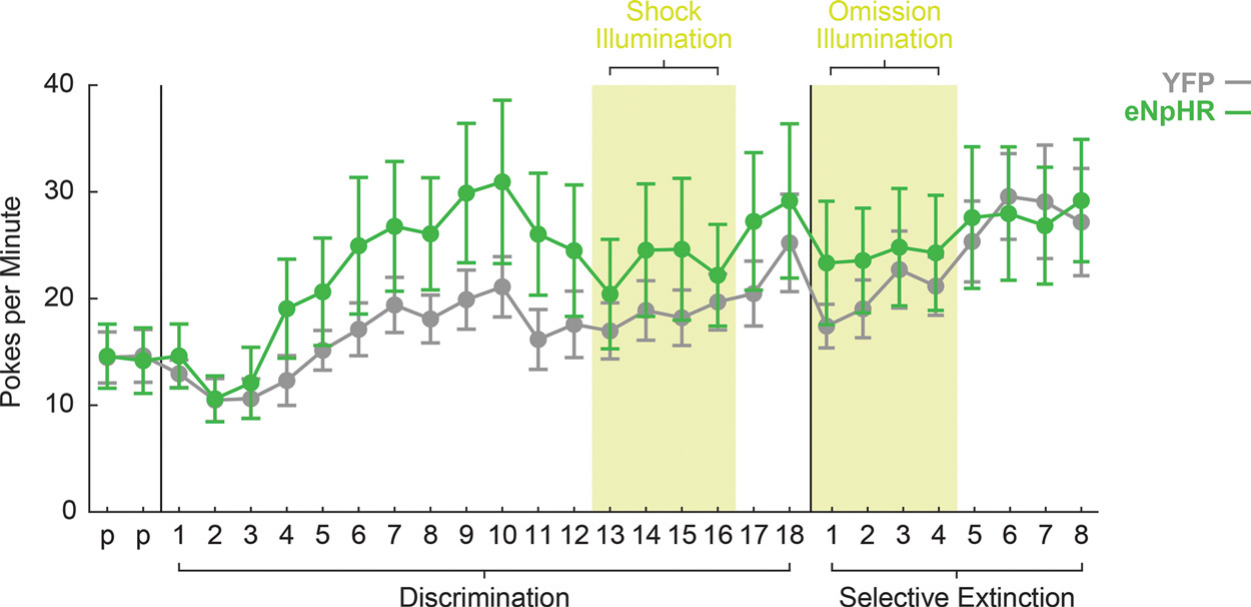
YFP versus eNpHR baseline nose poke rates. Mean ± SEM baseline nose poke rates for YFP (gray) and eNpHR rats (green) are plotted for the two preexposure (p), 18 discrimination, and eight selective extinction sessions. Rats were acclimated to the cables on discrimination sessions 11–12 by tethering to “dummy cables” without light illumination.

Repeated-measures ANOVA for baseline nose poke rate (within factor: session; between factors: sex and group) revealed main effects of session (*F*_(27,432)_ = 12.92, *p* < 0.001, η^2^p = 0.45, power = 1.00^d^), sex (*F*_(1,16)_ = 24.66, *p* < 0.001, η^2^p = 0.61, power = 0.99 ^d^), and group (*F*_(1,16)_ = 5.72, *p* = 0.029, η^2^p = 0.26, power = 0.61^d^). Interactions of group × sex (*F*_(1,16)_ = 7.62, *p* = 0.014, η^2^p = 0.32, power = 0.74^d^), group × session (*F*_(27,432)_ = 1.66, *p* = 0.021, η^2^p = 0.094, power = 0.99^d^), and session × sex (*F*_(27,432)_ = 3.53, *p* < 0.001, η^2^p = 0.18, power = 1.00^d^) also reached significance. eNpHR rats exhibited higher nose poke rates compared with YFP in preillumination fear discrimination sessions (Fig. 3). Sex effects were driven by higher poke rates in males compared with females, an effect consistent with previous behavioral findings in this paradigm (Walker et al., 2018, 2020). Importantly, there were no effects or interactions with group during either optogenetic manipulation period when these sessions were considered separately (all *F* < 2.87, all *p* > 0.05; Fig. 3).

#### Inhibition of DRN^tph2+^ neurons during +PE does not impact fear discrimination

**Figure 4.**
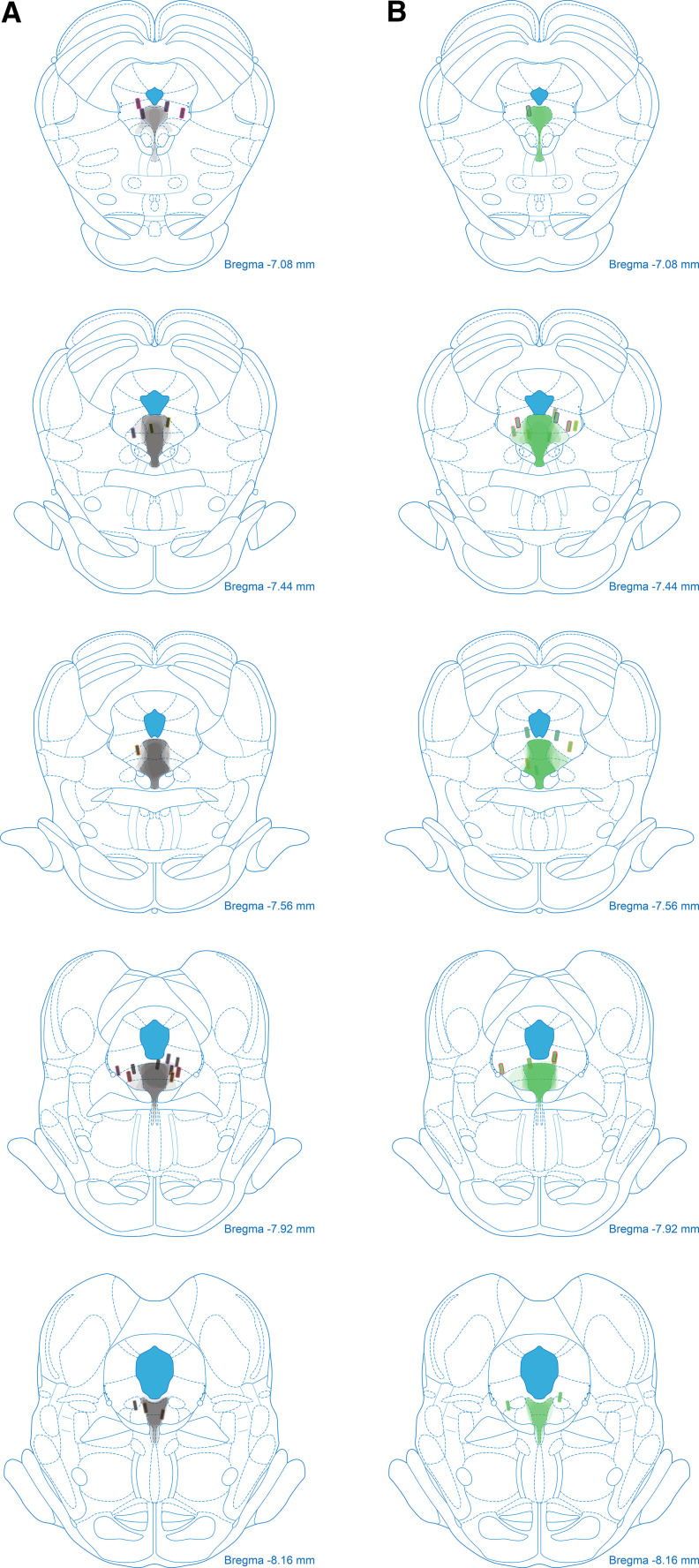
YFP and eNpHR viral expression and fiber placements. ***A***, Viral transfection extent is shown throughout the DRN for rats in the YFP (gray tracings) group. Bilateral ferrule placements (gray filled rectangles) for individual rats are denoted by colored outlines. ***B***, Viral transfections (green tracings) and ferrule placements (green filled rectangles) for eNpHR rats as in ***A***.

**Figure 5.**
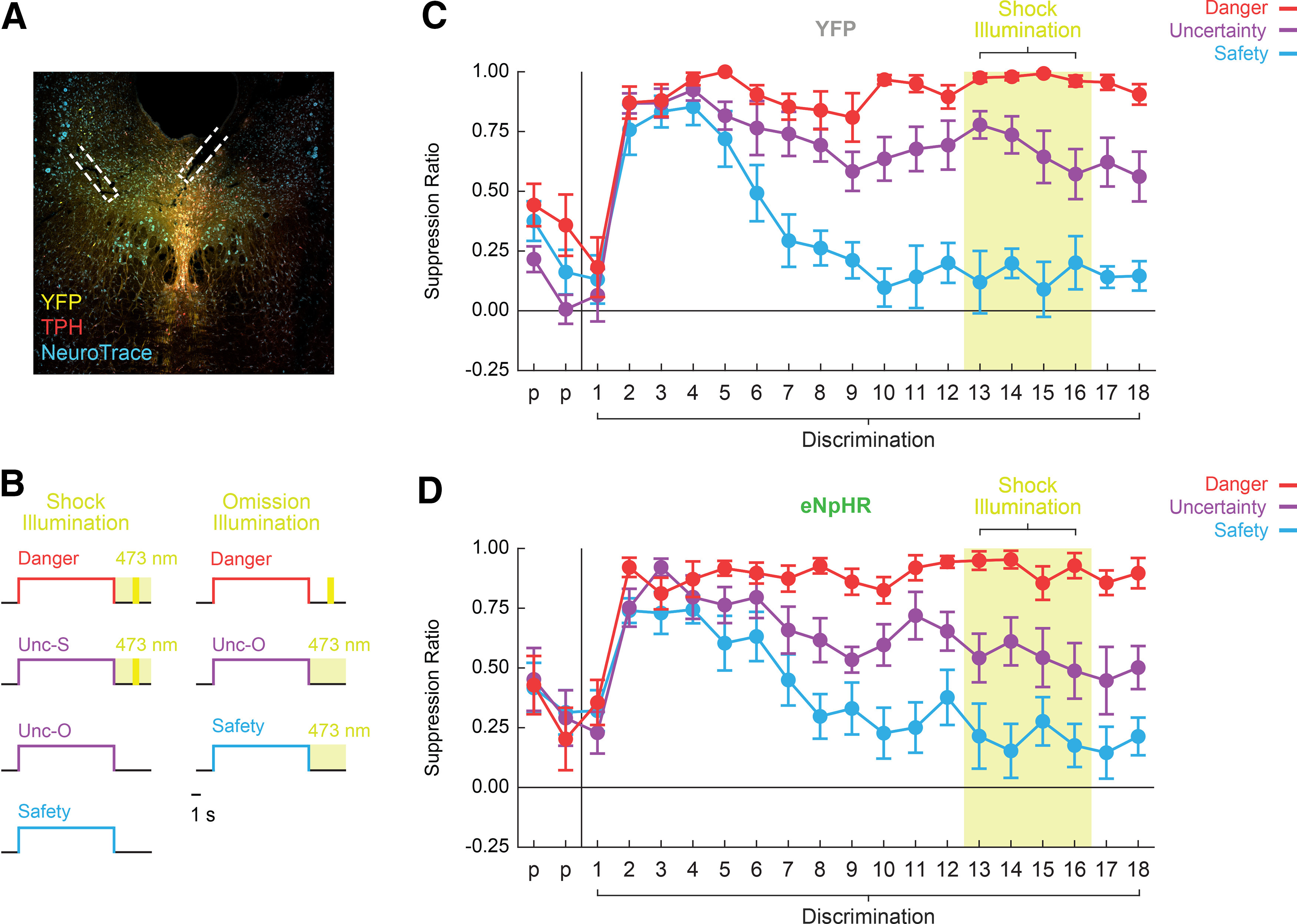
Inhibition of DRN^tph2+^ does not impact +PE-mediated fear discrimination. ***A***, Representative image shows ferrule implant sites (white dashes outlines) and viral transfection in DRN neurons (YFP, yellow) against tph2 immunohistochemistry (red) in a TPH2-cre rat. Fluorescent Nissl staining (NeuroTrace) shows neuronal cell bodies (blue). ***B***, During the +PE optogenetic manipulation, green-light illumination began at danger and uncertainty-shock cue offset, continued during the 0.5-s shock (yellow period), and lasted 1.5 s after shock for a total of 4 s. For the –PE optogenetic manipulation, green-light illumination began at uncertainty and safety cue offset for a total of 4 s. The uncertainty cue was selectively extinguished during these sessions. Note illumination never occurred during cue periods. ***C***, Mean ± SEM suppression ratios for YFP rats to danger (red), uncertainty (purple), and safety (blue) are shown for the two preexposure (p) and 18 discrimination sessions. Rats were acclimated to the cables on discrimination sessions 11–12 by tethering to “dummy cables” without light illumination. ***D***, eNpHR suppression ratio data shown as in ***C***. Both groups achieved discrimination, and light illumination during foot shock did not impact fear behavior.

TPH2-cre rats received bilateral DRN transfection with cre-dependent halorhodopsin or cre-YFP; all rats showed transfection in DRN^tph2+^ neurons with ferrules tips proximal to viral expression (Fig. 4; representative image in Fig. 5*A*). Rats were trained on the fear discrimination procedure before undergoing the +PE (Fig. 5*B*, left) and –PE (Fig. 5*B*, right) optogenetic manipulations. If DRN^tph2+^ neurons are involved in positive or unsigned errors, then inhibition of neural activity at the time of surprising foot shock (+PE manipulation) should alter fear to uncertainty, but have no effect on fear to danger.

Both YFP and eNpHR rats learned to discriminate between the three auditory cues, as indicated by significant effects of cue (*F*_(2,32)_ = 74.45, *p* < 0.001, η^2^p = 0.82, power = 1.00^e^), session (*F*_(13,208)_ = 40.23, *p* < 0.001, η^2^p = 0.71, power = 1.00^e^), and cue × session (*F*_(26,416)_ = 9.46, *p* < 0.001, η^2^p = 0.37, power = 1.00^e^). No effects of or interactions with group were found during preexposure or discrimination (all *F* < 0.97, all *p* > 0.05), indicating YFP (Fig. 5*C*) and eNpHR (Fig. 5*D*) rats showed equivalent levels of preillumination discrimination. There were no effects of sex during fear discrimination (all *F* < 2.29, all *p* > 0.05).

A lack of significant group effects during the +PE manipulation sessions (all *F* < 1.81, all *p* > 0.05^f^; Fig. 5*C*,*D*, green boxes), indicated light illumination during foot shock did not alter fear behavior in eNpHR rats. Again, there were no effects of sex during these sessions (all *F* < 1.71, all *p* > 0.05^f^). This pattern of behavior stayed consistent in the postillumination discrimination sessions, with no significant effects of group (all *F* < 3.14, all *p* > 0.05^g^). Thus, at the start of the –PE optogenetic manipulation, eNpHR and YFP rats showed equivalent fear discrimination.

#### Inhibition of DRN^tph2+^ neurons during –PE alters extinction of uncertainty

**Figure 6.**
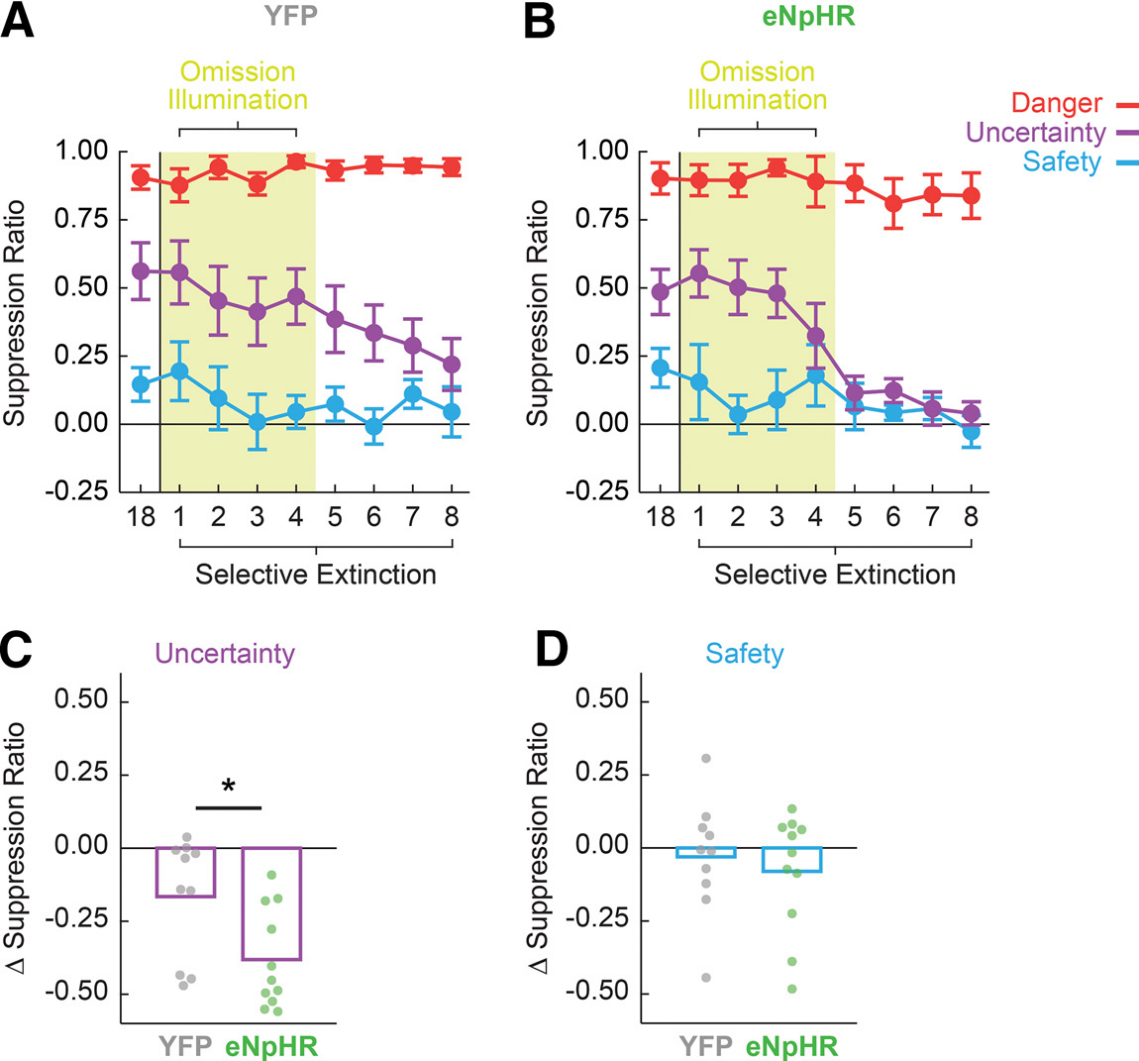
Inhibition of DRN^tph2+^ during –PE alters extinction of uncertainty. ***A***, Mean ± SEM suppression ratios for YFP rats to danger (red), uncertainty (purple), and safety (blue) are shown for the last fear discrimination (18) and eight selective extinction sessions. DRN illumination occurred during omission periods in sessions 1–4 for the –PE optogenetic manipulation. ***B***, eNpHR suppression ratio data shown as in ***A***. ***C***, Change in fear to the uncertainty cue between –PE optogenetics sessions and postillumination selective extinction sessions 5–8 for YFP (gray) and eNpHR (green) rats. Bars show group average and circles indicate individual difference scores *p < 0.05 between groups. ***D***, Change in fear to the safety cue as shown in ***C***.

A repeated-measures ANOVA for the uncertainty and safety cues revealed no significant effects of or interactions with group during the 4 –PE optogenetic sessions (all *F* < 1.51, all *p* > 0.05^h^), but main effects of group (*F*_(1,16)_ = 4.60, *p* = 0.048, η^2^p = 0.22, power = 0.52^h^) and sex (*F*_(1,16)_ = 6.57, *p* = 0.021, η^2^p = 0.29, power = 0.67^h^) emerged in the four no-illumination selective extinction sessions (Fig. 6*A*,*B*). Fear to danger did not change for either group during these sessions (all *F* < 1.17, all *p* > 0.05). These results indicated that DRN illumination did not impact fear behavior during illumination sessions, but instead produced a postillumination decrease in fear to uncertainty.

To better understand the behavioral change during and following illumination, difference scores were calculated for uncertainty and safety: (mean suppression ratio during the four postillumination sessions) – (mean suppression ratio during the four illumination sessions). For the uncertainty difference score, *t* test and bootstrap confidence intervals showed a significantly greater postillumination decrease in fear in the eNpHR group compared with YFP (*t*_(18)_ = 2.36, *p* = 0.030^i^; Fig. 6*C*). No group differences were observed for the safety difference score (*t*_(18)_ = 0.12, *p* = 0.90^j^; Fig. 6*D*).

To determine whether changes in fear occurred within-session or between-session, the first postillumination session was isolated, and six uncertainty cue trials were sampled during this session. While there was a significant main effect of group, reflecting the between-session effect above, there was no trial × group interaction (*F*_(5,90)_ = 0.57, *p* = 0.72, η^2^p = 0.031, power = 0.20^k^). These results reveal that reduced fear to uncertainty was present on the first trial and thus emerged between sessions, rather than within session. These results demonstrate that inhibition of DRN^tph2+^ neurons during omission periods did not immediately alter subsequent fear levels but rather impacts postinhibition fear to uncertainty. Additionally, the +PE manipulation ruled out DRN^tph2+^ involvement in positive or unsigned PE signaling.

## Discussion

In experiment 1, selective deletion of DRN^tph2+^ neurons resulted in slower and lesser fear extinction to the uncertainty cue. These results pointed to a possible deficit in –PE signaling, as these errors are used to decrease future fear. To then isolate PE functioning, in experiment 2, selective inhibition of DRN^tph2+^ neurons during shock and omission periods tested whether activity in these neurons were necessary for positive, negative, or unsigned PE signaling. There were no immediate effects of inhibition, yet fear to uncertainty significantly decreased in the sessions immediately following the –PE optogenetic manipulation. These findings are not consistent with the theoretical framework that DRN 5-HT neurons generate –PEs, instead they indicate that DRN^tph2+^ neurons are involved in –PE-mediated fear updating.

Taking a closer look at the implications of these results, experiment 1 demonstrated that deletion of DRN^tph2+^ neurons does not impact the ability to learn to discriminate between danger, uncertainty, and safety cues. These results were surprising because if DRN 5-HT is necessary to generate –PEs, as was initially hypothesized, then it would be expected that fear discrimination would be impacted. Results were consistent, however, with previous findings showing rats with global DRN neurotoxic lesions could acquire fear but were slower to extinguish fear to uncertain and danger cues (Berg et al., 2014). Given that the deletion occurred before any training and was not temporally tied to –PE periods, it was possible that a compensatory mechanism obfuscated a deficit from the lack of DRN^tph2+^ such that no behavioral differences could be seen during fear discrimination and only became visible during extinction of the uncertainty cue. It was thus essential to use a manipulation with the temporal resolution to target PE periods.

Experiment 2 first ruled out DRN^tph2+^ contribution to positive or unsigned PE signaling, as inhibition during shock and immediate postshock periods had to effect on behavior during +PE optogenetic manipulation sessions or the subsequent non-illumination discrimination sessions. While one explanation could be that learning to the uncertainty cue was at asymptote, and therefore no longer maintained by PE, previous experiments using the same task have demonstrated +PE generation and behavioral dependence on +PE even after extensive training (Walker et al., 2020). Additionally, behavioral results during the –PE optogenetic manipulation unexpectedly showed no impact of DRN^tph2+^ inhibition. If DRN 5-HT is necessary for –PE generation, inhibition during shock omission periods would be expected to have prevented decreases in fear to the uncertainty cue in eNpHR rats compared with YFP. Greater postoptogenetics extinction of uncertainty but no change in fear to safety suggested that while –PEs may still have been generated, DRN 5-HT plays a role in use of this error. As soon as DRN^tph2+^ signaling came back online, fear to uncertainty decreased, suggesting the neural representation of shock expectancy was indeed changing. Differences in fear to uncertainty also manifested between, not within sessions, providing further support to the idea that DRN^tph2+^ neurons are not computing –PE, only using it. The results of experiment 2 were unexpected and distinct from those of experiment 1, where DRN^tph2+^ deletion resulted in an extinction deficit. These opposing results are likely because of the nature of the manipulations, one permanent and the other brief manipulations of cellular activity, and their possible unintended consequences, namely possible compensatory mechanisms or alternate non-PE effects on fear learning because of permanent deletion of DRN^tph2+^ cells. Experiment 2 was indeed more optimally designed to target PE signals, so these results better speak to the contribution of DRN^tph2+^ cells to –PE updating.

DRN^tph2+^ contribution to –PE fear updating also does not appear to vary by sex. While there were a few significant behavioral differences between the sexes in experiments 1 and 2, none of the critical findings related to fear behavior were impacted by sex, indicated by a lack of sex × group or sex × group × session interactions. Baseline nose poke behavior was generally higher in males, but this effect is consistent with previous findings in the same task (Walker et al., 2018, 2020) and was not significant during optogenetics sessions. Overall, these findings support the idea that aversive PEs likely function similarly in males and females (Walker et al., 2020), but sex is still an important factor to consider in analyses, especially depending on the behavioral measurement.

If DRN 5-HT-expressing neurons do not generate, but rather receive, –PE signals then the question arises as to where the signal *is* generated. Based on the present findings, the source is likely to impact DRN serotonergic signaling, suggesting it may be a population synapsing onto DRN^tph2+^ neurons. This leaves open the possibility that this signal is generated elsewhere in the brain, but the experimental evidence presented here does not preclude the possibility than another DRN population could generate –PEs. DRN dopaminergic neurons are one such possibility. With direct projections to the lateral central amygdala (Matthews et al., 2016), DRN dopaminergic neurons would be well-positioned to influence the broader fear network and could interact with DRN 5-HT through local modulation. Additionally, molecular characterization of the DRN has indicated that DRN populations expressing 5-HT and DA are largely non-overlapping (Huang et al., 2019), indicating the manipulations of DRN^tph2+^ in the present experiments would not have interfered with DRN DA signaling. Despite the lack of overlap between 5-HT and DA, 5-HT is commonly co-expressed in the DRN with other transmitters and neuromodulators, particularly glutamate, substance P, CRF, and galanin (Michelsen et al., 2007). This is an important caveat for the present experiments, as manipulation of DRN^tph2+^ may have had unintended effects on non-5-HT neurotransmission. Finally, it should also be mentioned that recent evidence is revealing the brain’s 5-HT systems to be increasingly complex and distinct, with separable DRN^5-HT^ projections targeting specific regions and acting on different 5-HT receptors (Okaty et al., 2019). It is possible, and indeed likely, that these distinct systems gate disparate information, so manipulating distinct populations of DRN^5-HT^ cells, as opposed to the global DRN manipulations employed in these experiments, may produce more targeted changes in neural signaling and any corresponding behavior. Full characterization of manipulated cell groups will be necessary for future experiments to improve understanding of which neurotransmitters are responsible for experimental effects.

It is notable that a correlate for –PE (increased firing to shock omission on uncertainty trials) was not observed in vlPAG single-units during fear discrimination (Walker et al., 2020), indicating that vlPAG neurons only generate positive error signals unlike ventral tegmental area (VTA) DA neurons that can produce positively and negatively signed reward PEs (Schultz, 1997; Roesch et al., 2007). Interestingly, the VTA has recently been put forth as a potential source of aversive –PEs. VTA DA neurons have been shown to be activated by surprising shock omission, with fear extinction dependent on VTA DA (Salinas-Hernandez et al., 2018). Another group similarly demonstrated extinction dependence on VTA DA (Luo et al., 2018), and this population provides input to the DRN (Peyron et al., 2018). Further, DRN 5-HT neurons have been shown to express D1 and D2 DA receptors (Niederkofler et al., 2016; Okaty et al., 2019), indicating VTA inputs may even directly act on this population. VTA generation of –PE would be particularly interesting given its involvement in reward PEs. Causal evidence targeting the –PE period is still lacking, especially outside the context of extinction. Indeed, because the present and past findings were in the context of extinction, another interpretation of the present results may be that this effect is not because of prediction error, but rather an inhibitory learning process required for extinction. It is well established that extinction leads to the formation of a new inhibitory association, rather than unlearning of the original excitatory association. Nevertheless, there is evidence for multiple types of extinction, which may be largely dependent on the protocol employed (Goodman and Packard, 2019). Recent evidence has further demonstrated that extinction based on omission relies on the infralimbic cortex whereas extinction based on overexpectation employs the orbitofrontal cortex (Lay et al., 2020). In the present experiments, it is difficult to disentangle the relative contributions of prediction error and extinction given the –PE optogenetic manipulation occurred during extinction of the uncertainty cue but lack of reinforcement was also an occasional feature of the cue itself during conditioning. If DRN 5-HT neurons are required for new extinction learning, however, it would be surprising that DRN^tph2+^ optogenetic inhibition did not have an immediate impact on extinction during the four illumination sessions. Future experiments should target –PE in the absence of overt extinction to determine whether this effect is extinction based or prediction error based. *In vivo* single-unit recordings, in particular, would be ideally suited to differentiate between these processes because of the high temporal and cell-specific resolution of this method. Such testing is necessary to determine which, if any, of these populations generate –PEs.

Despite many questions surrounding aversive –PEs remaining unanswered, these experiments reveal DRN^tph2+^ as a node in –PE-mediated fear behavior and provide crucial insight to shape new hypotheses related to –PE signaling. Future studies are needed to determine whether other populations within the DRN or cells projecting onto DRN^tph2+^ cells, such as VTA dopaminergic neurons, might be involved in –PE signaling.

## Synthesis

Reviewing Editor: Laura Bradfield, University of Technology Sydney Decisions are customarily a result of the Reviewing Editor and the peer reviewers coming together and discussing their recommendations until a consensus is reached. When revisions are invited, a fact-based synthesis statement explaining their decision and outlining what is needed to prepare a revision will be listed below. The following reviewer(s) agreed to reveal their identity: Sydney Trask.

Thank you for your manuscript, the reviewers and I all found it to be interesting and a well-performed study. The reviewers have made a series of points for you to respond to, as included in the individual reviews below.

I would like to make a note with regards to the points made by reviewer #1 regarding the interpretation of prediction error, as we had some discussion on consultation. I agree with this reviewer that some consideration of an interpretation of the data regarding mechanisms other than prediction error should be considered and added to the manuscript. With regards to the comment about the term ‘positive prediction error’ being used incorrectly, it seems that the reviewer is suggesting that because the uncertainty cue is only ever paired with the footshock at a probability of .375, then associative learning that is commensurate with this probability IS asymptote for this cue. That is, if the danger cue that perfectly predicts the outcome has an associative strength of 1 at asymptote, the uncertainty cue has an associative strength of .375 at asymptote. Therefore, there is no positive prediction error for this cue when it is paired with footshock because this shock is perfectly predicted for the number of times it is expected to occur. This is a slightly different view to that presented by the authors, and one that ascribes a more static value to the uncertainty cue. I assume the authors were taking the view that the uncertainty cue continually undergoes updating when the footshock is or isn’t presented. Although I think that the term ’positive prediction error’ can remain here, I suggest that the authors insert some consideration into the manuscript of the fact that learning might be at asymptote for this cue (where asymptote = associative strength of .375) and thus there may not be positive prediction error after footshock following this cue, or that it might be negligible.

Please also do be sure to address the points of reviewer #2, especially that regarding the manipulation of neurons that express 5-HT versus manipulating 5-HT itself.

The specific reviews are attached:

Reviewer #1

The present manuscript describes two experiments in which the authors investigate how DRN 5HT impacts extinction learning. In a first experiment, rats were given fear discrimination training paradigm in which one cue was never paired with the unconditional stimulus (what they term a ’safety’ cue), one is always paired with the UCS (’danger’ cue), and one is only presented sometimes (with a probability of 0.375; ’uncertainty’ cue) following selective deletion of 5HT neurons in the DRN. Then, the rats underwent selective extinction of the uncertainty cue. While there was no impact of DRN 5HT deletion in the acquisition of the discrimination paradigm, it did slow later extinction to the uncertainty cue. A second experiment aimed to examine how optogenetic inhibition of these neurons during the time of the expected shock would impact extinction. Contrary to the results of the first experiment, optogenetic inhibition of DRN 5HT neurons during the time of expected shock resulted in enhanced extinction, observable starting the fourth session of optogenetic manipulation during extinction and lasting throughout the rest of the light-free extinction sessions.

Overall, the manuscript is well-written and asks an interesting question about how DRN 5HT neurons might influence extinction learning. I have several major concerns with the interpretation and believe the manuscript could be substantially overhauled to reflect that this is an extinction phenomenon that may or may not be governed solely by prediction error. These are outlined in more detail below.

Major Comments:

1) The manuscript is currently written entirely as a prediction error paper, despite the main variable of interest being extinction learning. While prediction error likely contributes to extinction, especially in the early phases, extensive research has demonstrated that extinction is not just an updating of an originally learned CS-UCS association (as would be predicted by quantitative models of learning, like the Rescorla-Wagner (1972) model). Instead, many people have spent entire careers demonstrating that extinction results in new, inhibitory learning. The present manuscript attributes all of the observed extinction behavior (or lack thereof, as in Experiment 1) to deficits in error-mediated updating, when instead a more parsimonious explanation of the present results would be to couch the discussion in terms of inhibitory learning that is acquired through extinction. If the authors are interested in how DRN 5HT affects prediction error, I would suggest doing so in the absence of overt extinction (for example, using an overexpectation design). Much of the present introduction and discussion need to be substantially reworked to convey that the present experiments are examining extinction learning rather than prediction error.

2) The authors describe an optogenetic manipulation aiming to assess positive prediction error (see Fig 4D, E). I am unsure how this, as written and presented in the figure, assesses positive prediction error at all as the animals already seem to be conditioned to asymptote. As written and presented, ’positive prediction error’ is a misnomer here. Instead, the authors might have meant this as a control to contrast how their optogenetic manipulation impacts late acquisition sessions compared to extinction, but I’m not sure. The procedure should either be clarified or any indication that this is positive prediction error should be removed.

3) Using different approaches to target DRN 5HT in Experiments 1 and 2, the authors find opposite results (impairments in extinction learning in Experiment 1 and facilitation of extinction learning in Experiment 2). The discussion should explain these differences as well as why they might have occurred.

4) Related to my major point 1, the present manuscript consistently refers to extinction as fear updating through prediction error. I would suggest removing this terminology as memory updating refers to a different process dependent on reconsolidation-like mechanisms that are distinct from extinction (which as stated above relies on acquisition of a new, inhibitory memory).

Minor Comments:

1) Line 40 - The authors say negative prediction error occurs when the actual outcome is ‘worse’ than the expected outcome. The word worse is subjective, and this sentence is itself not necessarily true. Instead, I believe the authors meant to say that the actual outcome is reduced relative to the expected outcome. In a fear extinction paradigm, the case could be made that the rat perceives the actual outcome, no shock, as BETTER than the expected shock outcome, not worse.

2) Line 127 - I am assuming vlPAG is a typo and the authors meant instead DRN?

3) Might be a formatting issue, but the figure axes all have staggered labels that are difficult to read. Please disregard if this was something that got lost in translation on the conversion to PDF for the submission site.

Reviewer #2

Overall, this is an interesting and well performed study. There are no experimental issues that need to be directly addressed, however there are some areas that are lacking. Specifically, I think the words used to describe the experiments and the results, are at times, incorrect.

1) The authors do not ever specifically manipulate 5HT in this manuscript. They are manipulating neurons that express 5HT. But notably, these neurons express a host of other transmitters and modulators. This is an essential aspect of the study that is not brought up in the abstract. It needs to be. There are many instances of this same lack of clarity in the manuscript. As such, there is no discussion of the role of potential co-transmitters. This is an important confound.

one example:

"Rats in the Caspase group showed robust deletion of 5-HT throughout the DRN,

286 while rats in the YFP group showed 5-HT intact, as verified by anti-tph2

287 immunohistochemistry (Fig. 2A, B).”

-In this instance, the authors are not measuring 5HT. they are measuring a marker for 5HT containing neurons. language should be corrected.

2) There did not appear to be any quantification of the extent of lesion with caspase, or of any of the infections or a detailed description of where the fibers were placed. This should be included, as well as correlated with outcomes.

3) There is also no discussion of the concept that subsets of DRN 5HT output neurons may be gating different information. In that same vein, there is a well known anatomical segregation of 5HT outputs, based on the lack of detail regarding placement of fibers and map of expression, it is difficult to integrate these studies in to the larger view of 5HT.
